# Stage-dependent angiopoietin-Tie2 and nitric oxide signaling of erythrocytes in response to surgical trauma in head and neck cancer

**DOI:** 10.1186/s12957-020-01991-9

**Published:** 2020-08-16

**Authors:** Hsiang-Ling Wu, You-Hsiang Chu, Ying-Hsuan Tai, Mei-Yung Tsou, Cheng-Hsien Wu, Wen-Liang Lo, Shyh-Kuan Tai, Chun-Chang Yeh, Chih-Cherng Lu

**Affiliations:** 1 Department of Anesthesiology, Taipei Veterans General Hospital, No. 201, Sec. 2, Shih-pai Rd, Taipei, 11217 Taiwan; 2 School of Medicine, National Yang-Ming University, Taipei, Taiwan; 3 Graduate Institute of Life Sciences, National Defense Medical Center, Taipei, Taiwan; 4 Department of Anesthesiology, Shuang Ho Hospital, Taipei Medical University, New Taipei City, Taiwan; 5 Department of Anesthesiology, School of Medicine, College of Medicine, Taipei Medical University, Taipei, Taiwan; 6 Division of Oral and Maxillofacial Surgery, Department of Stomatology, Taipei Veterans General Hospital, Taipei, Taiwan; 7 Department of Otolaryngology, Taipei Veterans General Hospital, Taipei, Taiwan; 8 Department of Anesthesiology, Tri-Service General Hospital and National Defense Medical Center, No. 325, Sec. 2, Chenggong Rd., Neihu Dist., Taipei, 114 Taiwan; 9 Institute of Aerospace Medicine, National Defense Medical Center, Taipei, Taiwan

**Keywords:** Angiogenesis, Carcinoma, Nitric oxide, Surgical resection, Surgery

## Abstract

**Background:**

Angiopoietin-Tie2 and nitric oxide pathway is crucial in tumor angiogenesis and closely correlates with tumor development, growth, and metastasis. This study aimed to investigate the angiopoietin-Tie2 and nitric oxide signaling of the erythrocyte membrane in response to surgical trauma in head and neck cancer.

**Methods:**

We prospectively enrolled the patients with histology-proven head and neck squamous cell carcinoma undergoing surgical resection of primary tumors at the medical center between August and November 2019. We measured the preoperative and postoperative levels of angiopoietin-1, angiopoietin-2 in plasma using enzyme-linked immunosorbent assays, nitric oxide in plasma using nitrate/nitrite colorimetric assays, and Tie2 phosphorylation in erythrocyte membrane using Western blotting.

**Results:**

The plasma angiopoietin-1 was downregulated from the median 971.3 pg/mL (interquartile range [IQR] 532.1–1569.3) to 417.9 (IQR 270.5–597.3) after tumor resection (*p* = 0.0020). Conversely, the plasma angiopoietin-2 was enhanced from 1173.6 pg/mL (IQR 977.7–1450.2) to 2353.7 (IQR 1352.4–2954.3) after surgery (*p* = 0.0021), with a concomitant increase in plasma nitric oxide level from 7.73 μM (IQR 5.39–10.06) to 10.50 (IQR 7.65–14.18) after surgical resection (*p* = 0.0093). Subgroup analyses further showed the angiopoietin-Tie2 and nitric oxide signaling was significant only in stage III and IV cancer.

**Conclusions:**

The dynamic change of angiopoietin-Tie2 signaling in the erythrocyte membrane along with the enhanced nitric oxide in plasma after tumor resection suggests erythrocytes play a significant role in modulating surgery-induced angiogenesis, which may provide a novel marker for cancer surveillance and control.

## Background

Over 500,000 new cases of head and neck squamous cell carcinoma (HNSCC) were diagnosed worldwide each year, and two thirds of them occurred in industrialized countries [1]. In HNSCC, carcinogenesis is a two-step process featured by an initial precancerous lesion and the malignant transformation characterized by an angiogenic switch with an increase in neovascularization [2].

Angiogenesis, the process through which new blood vessels form from pre-existing vessels, is a critical element of tumor growth and metastasis in HNSCC as in other solid tumors [3]. The angiopoietin-Tie2 signaling pathway is crucial in regulating tumor angiogenesis and closely linked to the development, progression, and metastasis of cancer cells [4–6]. The regulation of the cytokine angiopoietin-1 and angiopoietin-2 functions in angiogenesis and vascular remodeling through their interaction with the vascular receptor tyrosine kinase Tie2 [7]. Previous studies have showed that expression of angiopoietin-2 was significantly associated with angiogenesis and vessel maturation in oral squamous cell carcinoma (SCC) [6]. An animal study reported that overexpression of angiopoietin-2 has also been shown to promote the growth and metastasis of oral SCC [8]. Besides, an experimental study demonstrated the distinct expressions of angiopoietin-1 and angiopoietin-2 in tumor samples of human oral SCC [9]. However, the dynamic changes of these angiogenic factors in the setting of tumor resection remains unclear.

Nitric oxide (NO) is essential in various physiological as well as pathological processes. In recent decades, NO was demonstrated to regulate different cancer-related events including angiogenesis, progression, and metastasis [10]. NO may induce and promote tumor angiogenesis through the mechanism of vessel dilatation by endothelial NO synthase (eNOS), release of vascular endothelial growth factor (VEGF), and increase the production of prostaglandin E2 inducing tumor vasculature hyperpermeability [10].

Although surgical resection is a potential curative for patients with HNSCC tumors, a surgical injury may trigger the release of proangiogenic factors and facilitate the growth and spread of residual cancer cells [1, 11]. Prior studies have showed that plasma levels of angiopoietin-2 and VEGF were increased after surgical resection of primary tumors in the lung, breast, and colorectal cancer [11, 12]. However, the angiopoietin-Tie2 and NO signaling in response to surgical injury remain unclear in HNSCC.

Erythrocytes act as an important interorgan communication network in controlling systemic NO metabolism and tissue oxygen transport [13]. Our previous study showed the hypo-osmotic stimulus enhances angiopoietin-1 secretion in plasma through the Tie2/Akt/eNOS signaling pathway in erythrocytes [14]. However, the role of erythrocytes in cancer angiogenesis has not been clarified yet.

Based on the above background, this study aimed to evaluate the angiopoietin-Tie2 and NO signaling in the erythrocyte membrane in response to surgical treatments of HNSCC. Specifically, we conducted this study by measuring the dynamic changes in plasma angiopoietin-1, angiopoietin-2, and NO levels and determined the phosphorylation level of tyrosine kinase receptor Tie2 in erythrocyte membrane before and after surgical resection of HNSCC.

## Methods

### Clinical setting and patient enrollment

We enrolled the patients with histology-proven HNSCC undergoing surgical resection of primary tumors with or without node dissection and flap reconstruction at the medical center between August and November 2019. Exclusion criteria were aged below 20 years, any history of erythrocyte disorder (e.g., sickle cell disease, glucose-6-phosphate dehydrogenase deficiency), uses of exogenous NO provider (e.g., glycerin trinitrate or sodium nitroprusside), prior surgery for malignancy or other pathologies in head and neck region, recurrent diseases of HNSCC, and patient refusal to participate. After meeting the selection criteria, a total of 14 patients with HNSCC were enrolled. In order to compare the baseline levels of angiopoietin-Tie2 and NO signaling, we included 14 volunteers without any history of malignancy, who were regarded as non-cancer controls.

### Study outcomes

The primary outcome of this study was the plasma concentrations of angiopoietin-1 and angiopoietin-2 measured before and after surgical resection. Secondary outcomes comprised the plasma levels of NO and the tyrosine phosphorylation level of Tie2 in the erythrocyte membrane.

### Collection of covariates

We collected the data of patient characteristics, including demographics, American Society of Anesthesiologists class, and habits of tobacco smoking, alcohol intake, and betelnut chewing [15, 16]. Clinical covariates included types of surgical procedures, anesthesia time, intraoperative blood loss, and intraoperative uses of blood transfusion [17, 18]. Pathology features were differentiation grade, angiolymphatic invasion, and perineural invasion [19, 20]. Tumor nodes metastasis (TNM) staging was translated into stages I to IV according to the American Joint Committee on Cancer (AJCC) criteria, the seventh edition [21].

### Blood sample collection

The peripheral blood was sampled through radial arterial catheters before surgical incision (baseline) and at the end of surgery. Blood samples were stored in K2 ethylenediaminetetraacetic acid (EDTA)-containing tubes (Becton Dickinson, NJ, USA) for plasma and protein extraction. Plasma was centrifuged at 3000 rpm for 10 min without brake and then stored at − 80 °C. Erythrocytes were isolated by Ficoll Paque Plus (GE Healthcare Bio-Sciences AB, Uppsala, Sweden). Equal amounts of erythrocytes were added in three-volume times of ice cold 1 mM Na_2_HPO_4_, 1 mM EDTA, 1 mM phenylmethylsulfonyl fluoride (PMSF, Gold Biotechnology, St. Louis, MO, USA), and pH 7.4 wash buffer. The erythrocyte membrane was washed to eliminate hemoglobin by pelleting at 14,500 rpm for 20 min at 4 °C for over four wash cycles and then also stored at − 80 °C for further processing.

### Nitric oxide detection

NO concentration in plasma was measured by determining its stable end products, nitrite and nitrate [22]. Plasma nitrite and nitrate were measured by the nitrate/nitrite colorimetric assay kit (Cayman Chemical®, MI, USA). The first step was the conversion of nitrate to nitrite using nitrate reductase. The second step was the addition of the Griess reagents. After 10 min of incubation at room temperature, the absorbance is measured at 550 nm wavelength with a microplate reader (BioTek Instruments, VT, USA).

### Angiopoietin-1 and angiopoietin-2 expression

To detect the levels of angiopoietin-1 and angiopoietin-2 in plasma, enzyme-linked immunosorbent assay (ELISA) kits were used and carried out according to the manufacturer’s protocol (DANG10 and DANG20, R&D, MN, USA). Briefly, standards of angiopoietin-1 and angiopoietin-2 were prepared, and 1:5 diluted plasma samples were pipetted into wells with specific monoclonal antibody pre-coated microplate. After washing away unbound solution, enzyme-linked monoclonal antibodies were added to wells. Following a wash to remove any unbound antibody-enzyme solutions, a substrate solution was added to wells and the color developed in proportion to the control or sample concentrations. Finally, a tetramethylbenzidine solution was added to stop the reaction. We detected the intensity of the reaction color at 450 nm wavelength with a microplate reader (BioTek Instruments, VT, USA). All measurements were performed in duplicate.

### Immunoprecipitation for Western blotting

The level of Tie2 tyrosine phosphorylation was detected by an immunoprecipitation assay. The method for Tie2 tyrosine phosphorylation immunoprecipitation was described in the authors’ previous articles [14, 23]. Specific antibody for detecting total Tie2 was purchased from Abcam (Abcam, Cambridge, UK). β-actin was purchased from SantaCruz (Santa Cruz Biotechnology, TX, USA). Specific bands from an immunoblotting reaction were visualized using the Enhanced Chemiluminescence System (Millipore, MA, USA). The intensity of each band was analyzed using the ImageJ software (National Institutes of Health, Bethesda, MD, USA).

### Statistical analysis

A sample size of 14 was estimated to have 80% power to detect an effect size of 80% change in angiopoietin-1 or angiopoietin-2 levels in plasma for paired difference comparisons with a 2-sided significance level of 0.05 [24]. Shapiro-Wilk test and Kolmogorov-Smirnov test were used as normality tests. Normally distributed variables were presented as the mean with standard deviation (SD). Non-normally distributed data were presented as median with interquartile range [IQR] and range. Independent *t* test or Mann-Whitney *U* test was used to compare cancer patients and non-cancer controls in outcome variables before surgery, as appropriate. Paired sample *t* test or Wilcoxon signed-rank test was used to determine the statistical difference in changes of angiopoietin-1, angiopoietin-2, and NO levels in plasma and Tie2 phosphorylation levels before and after surgery in the cancer patients, as appropriate. The significance level of all hypotheses was 0.05 for a two-sided test. Statistical analyses were conducted using SAS software, version 9.4 (SAS Institute Inc., Cary, NC, USA) and presenting graph with Prism version 5.0 (GraphPad Software, San Diego, CA, USA).

## Results

Table 1 showed the demographic data, cancer characteristics, and type of surgical procedures of the cancer patients. In cancer characteristics, 7 out of the 14 cancer patients had stage IV diseases. Besides, 7 and 9 patients had the histology diagnosis of angiolymphatic invasion and perineural invasion, respectively. Regarding the surgical procedures, 12 patients underwent neck dissection for the removal of lymph nodes and surrounding tissue from the neck, and 12 patients underwent flap reconstruction. Table 2 showed the intraoperative hemodynamic and biochemical parameters. The anesthesia time was mean 790 ± SD 285 min. Intraoperative blood loss was 500 ± 366 mL, and 6 patients needed intraoperative transfusions of the blood.

**Table 1:** Demographic data, cancer characteristics, and type of surgical procedures of the cancer patients

	Cancer patients (*n* = 14)
Age, years	58.3 ± 8.8
Sex, male	11 (78.6)
Body weight, kg	71.4 ± 9.3
Body height, cm	166.7 ± 6.5
Body mass index, kg/m^2^	25.6 ± 2.3
ASA class	
II	6 (42.9)
III	8 (57.1)
Tobacco smoking	8 (57.1)
Alcohol intake	7 (50.0)
Betelnut chewing	9 (64.3)
Primary tumor site	
Lip	1 (7.1)
Tongue	2 (14.3)
Buccal mucosa	4 (28.6)
Gingiva	3 (21.4)
Palate	2 (14.3)
Hypopharynx	1 (7.1)
Larynx	1 (7.1)
TNM classification	
T1	3 (21.4)
T2	4 (28.6)
T3	1 (7.1)
T4	6 (42.9)
N0	9 (64.3)
N1	1 (7.1)
N2	0 (0)
N3	4 (28.6)
M0	14 (100.0)
Cancer stage	
I	3 (21.4)
II	2 (14.3)
III	2 (14.3)
IV	7 (50.0)
Differentiation grade	
Good	1 (7.1)
Moderate	10 (71.4)
Poor	3 (21.4)
Angiolymphatic invasion	7 (50.0)
Perineural invasion	9 (64.3)
Preoperative chemotherapy	1 (7.1)
Type of surgical procedures	
Neck dissection	12 (85.7)
Flap reconstruction	12 (85.7)

Values were mean ± standard deviation or counts (percent)

*ASA* American Society of Anesthesiologists

**Table 2:** Intraoperative hemodynamic and biochemical parameters of the cancer patients

	Before surgery	End of surgery
Systolic blood pressure, mmHg	147 ± 22	148 ± 32
Diastolic blood pressure, mmHg	90 ± 12	82 ± 22
Mean blood pressure, mmHg	109 ± 12	104 ± 22
Heart rate, beats/min	75 ± 12	96 ± 14
Body temperature, °C	36.1 ± 0.8	36.4 ± 1.1
SpO_2_, %	98 (96–99)	100 (98–100)
Serum glucose, mg/dL	114 (100–142)	141 (118–155)
Hemoglobin, g/dL	13.4 ± 0.9	11.8 ± 1.0
	Intraoperative parameters	
Anesthesia time, min	790 ± 285	
Surgical blood loos, mL	500 ± 366	
Blood transfusion		
Red blood cells	5 (35.7)	
Fresh frozen plasma	1 (7.1)	

Values were mean ± standard deviation, counts (percent), or median (interquartile range)

*SpO*_*2*_ oxyhemoglobin saturation by pulse oximetry

### Baseline levels of plasma angiopoietin-1 and angiopoietin-2

The plasma level of angiopoietin-1 was significantly higher in cancer patients compared to controls, median 671.4 pg/mL (IQR 440.9–1035.9, range 359.1–2490.4) vs. 288.9 (IQR 189.8–389.6, range 98.4–492.9) (Mann-Whitney *U* test, *p* = 0.0028; Shapiro-Wilk test, *p* < 0.0001; Kolmogorov-Smirnov test, *p* < 0.0100). The plasma level of angiopoietin-2 was lower in cancer patients compared to controls, median 1362.9 pg/mL (IQR 1223.1–1707.7, range 750.4–2054.2) vs. 1868.5 (IQR 1531.0–2338.4, range 1248.7–5489.9) (Mann-Whitney *U* test, *p* = 0.0257; Shapiro-Wilk test, *p* < 0.0001; Kolmogorov-Smirnov test, *p* < 0.0100).

### Baseline level of Tie2 tyrosine phosphorylation in the erythrocyte membrane

**Fig. 1:**
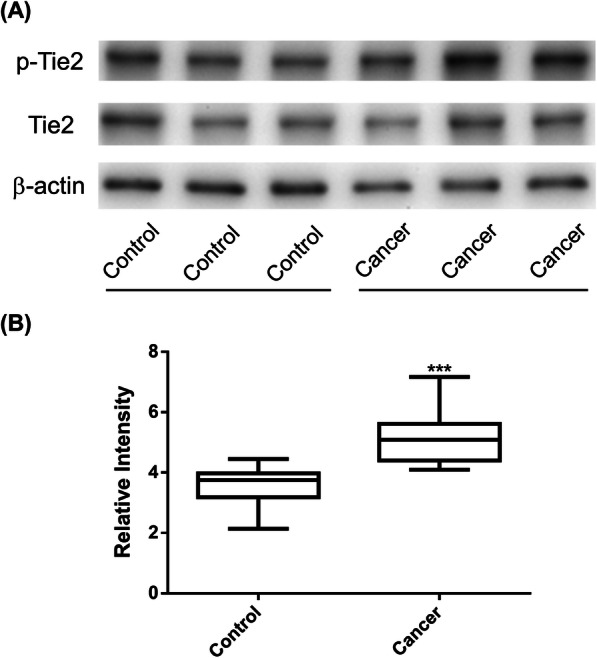
Tie2 tyrosine phosphorylation assay in the erythrocyte membrane. **a** A representative gel was shown. **b** Semi-quantitative analysis with ratio of the intensity of phospho-Tie2 receptor protein relative to total Tie2 receptor protein and β-actin indicated. *n* = 10, ***p < 0.001

The intensity ratio of phospho-Tie2/Tie2/β-actin in the erythrocyte membrane was significantly higher in cancer patients compared to controls, mean 5.18 ± SD 0.92 vs. 3.55 ± 0.68 (independent *t* test, *p* = 0.0003; Shapiro-Wilk test, *p* = 0.8392; Kolmogorov-Smirnov test, *p* > 0.1500) (Fig. 1).

### Baseline level of plasma nitric oxide

The plasma NO level of cancer patients was significantly higher than non-cancer controls, median 4.95 μM (IQR 2.70–11.90, range 1.30–19.00) vs. 1.42 (IQR 1.13–2.75, range 0.64–5.69) (Mann-Whitney *U* test, *p* = 0.0022; Shapiro-Wilk test, *p* < 0.0001; Kolmogorov-Smirnov test, *p* < 0.0100).

### Dynamic change of plasma angiopoietin-1 and angiopoietin-2 during surgery

**Fig. 2:**
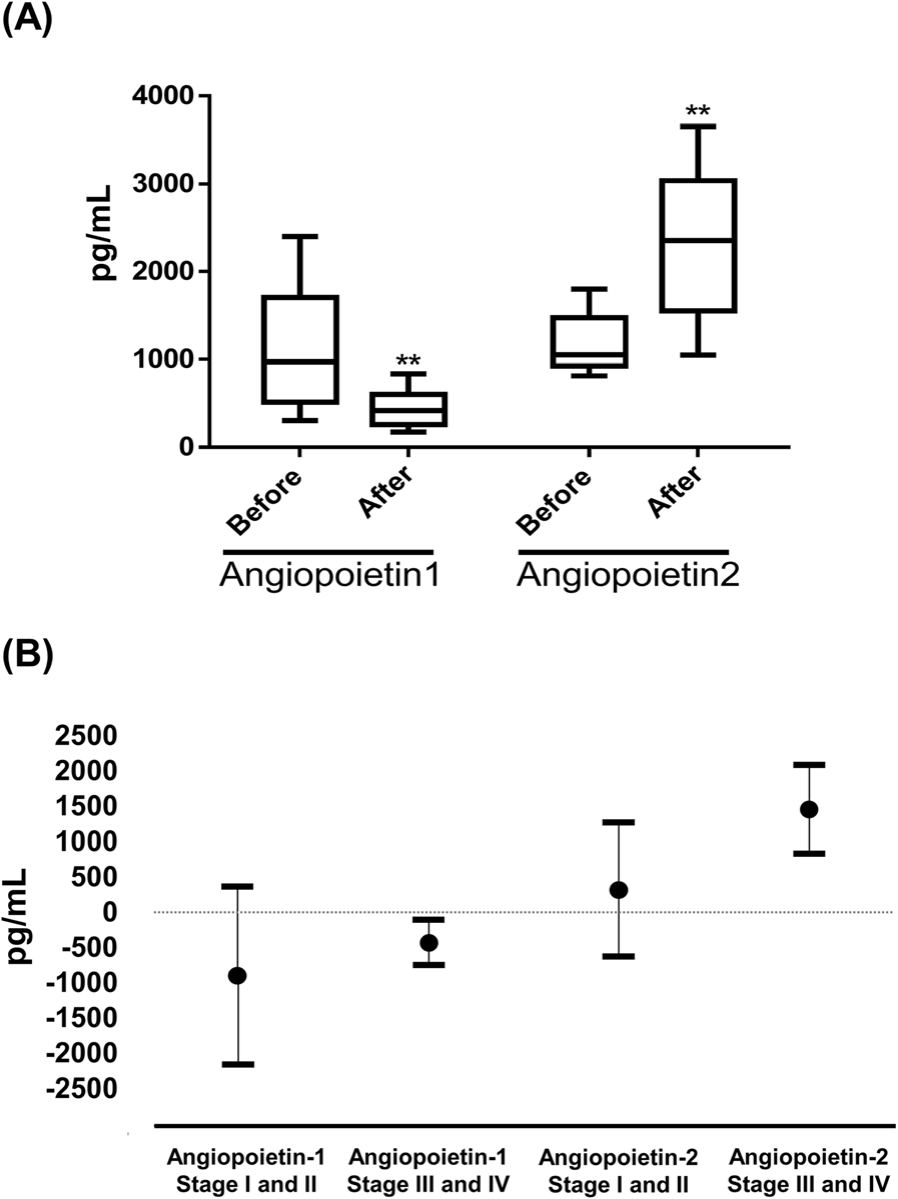
**a** Angiopoietin-1 and angiopoietin-2 plasma levels before and after surgery in cancer patients; **b** change in angiopoietin-1 and angiopoietin-2 levels, mean and 95% confidence interval. *n* = 10, **p < 0.01

The plasma level of angiopoietin-1 was downregulated from median 971.3 pg/mL (IQR 532.1–1569.3, range 303.7–2400.7) to 417.9 (IQR 270.5–597.3, range 176.0–839.1) after tumor resection [change: mean − 638.8, 95% confidence interval (CI), − 989.3 to − 288.3; paired sample *t* test, *p* = 0.0020; Shapiro-Wilk test, *p* = 0.1301; Kolmogorov-Smirnov test, *p* > 0.1500]. Conversely, the plasma level of angiopoietin-2 was significantly upregulated from median 1173.6 pg/mL (IQR 977.7–1450.2, range 813.7–1802.1) to 2353.7 (IQR 1352.4–2954.3, range 791.1–3653.4) after surgery (change 970.2, 95% CI 435.3–1505.0; paired sample *t* test, *p* = 0.0021; Shapiro-Wilk test, *p* = 0.9899; Kolmogorov-Smirnov test, *p* > 0.1500) (Fig. 2a).

Subgroup analyses showed the baseline level of plasma angiopoietin-1 was median 736.6 pg/mL (IQR 496.5–1177.7, range 303.7–1839.7) in stage III and IV cancer and 1299.0 pg/mL (IQR 1224.2–1844.0, range 402.4–2400.7) in stage I and II cancer (Mann-Whitney *U* test, *p* = 0.1439). The baseline level of plasma angiopoietin-2 was median 1261.8 pg/mL (IQR 1018.6–1579.6, range 967.5–1802.1) in stage III and IV cancer and 987.9 pg/mL (IQR 883.9–1303.8, range 813.7–1499.1) in stage I and II cancer (Mann-Whitney *U* test, *p* = 0.2556). The decrease in angiopoietin-1 and increase in angiopoietin-2 after surgery were significant only in stage III and IV diseases [change of angiopoietin-1: mean − 430.8 (95% CI − 750.3 to − 111.3, *p =* 0.0164) in stage III and IV and − 903.0 (95% CI − 2165.9–359.9, *p =* 0.1074) in stages I and II; change of angiopoietin-2: 1456.5 (95% CI 832.3–2080.7, *p =* 0.0012) in stages III and IV and 320.2 (95% CI − 628.9–1269.4, *p =* 0.3616) in stages I and II] (Fig. 2b).

### Dynamic change of Tie2 tyrosine phosphorylation during surgery

**Fig. 3:**
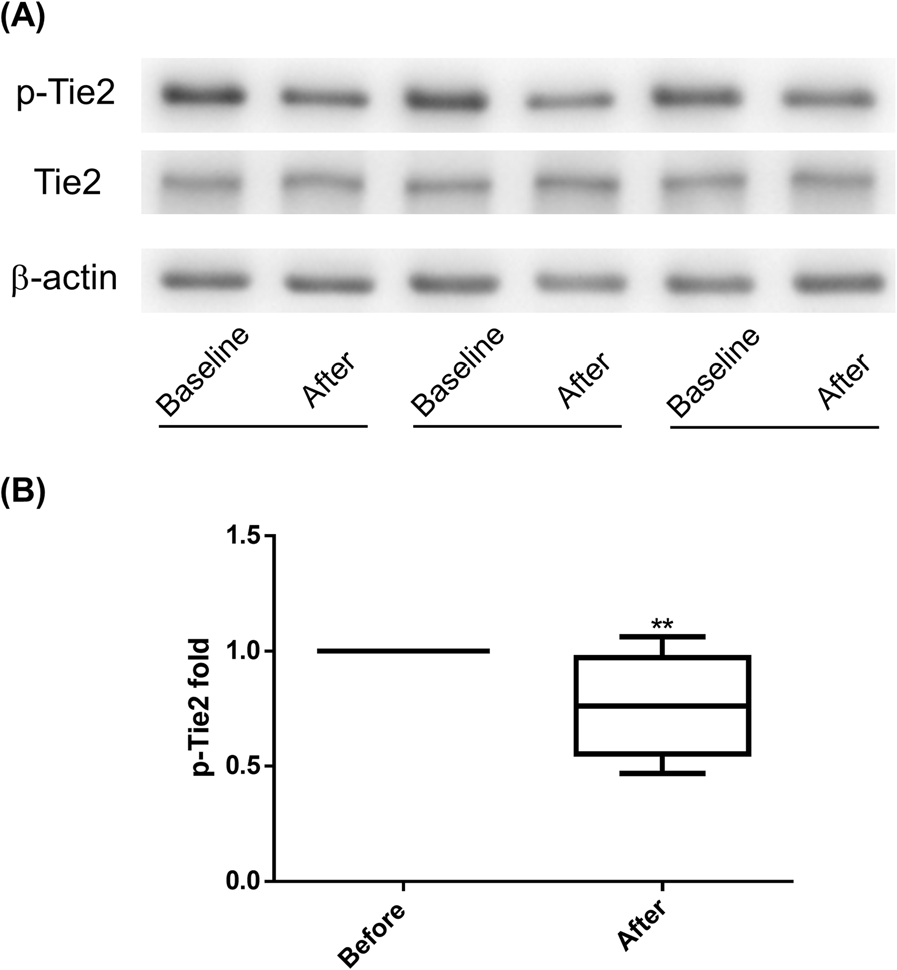
Comparisons of Tie2 phosphorylation levels in the erythrocyte membrane before and after surgery in cancer patients. **a** A representative gel was shown. **b** Intensity ratio of Tie2 receptor protein tyrosine phosphorylation relative to total Tie2 receptor protein. Tie2 tyrosine phosphorylation level was significantly downregulated after surgery in cancer patients. *n* = 10, **p < 0.01

The level of Tie2 tyrosine phosphorylation in the erythrocyte membrane was significantly decreased in cancer patients after surgery with a relative change − 23.2 ± 21.2% (paired-sample *t* test, *p* = 0.0072; Shapiro-Wilk test, *p* = 0.5501; Kolmogorov-Smirnov test, *p* > 0.1500) (Fig. 3).

### Dynamic change of plasma nitric oxide during surgery

The level of plasma NO was significantly enhanced from median 7.73 μM (IQR 5.39–10.06, range 1.84–18.58) to 10.50 (IQR 7.65–14.18, range 2.49–33.11) after surgical resection (Wilcoxon signed-rank test, *p* = 0.0093; Shapiro-Wilk test, *p* = 0.0009; Kolmogorov-Smirnov test, *p* < 0.0100).

The baseline level of plasma NO was median 9.45 μM (IQR 7.19–12.39, range 4.66–18.58) in stage III and IV cancer and 6.11 μM (IQR 2.29–6.46, range 1.84–8.81) in stage I and II cancer (Mann-Whitney *U* test, *p* = 0.0513). Similarly, subgroup analyses showed the enhancement of plasma NO was significant only in stage III and IV diseases [median 9.45 (IQR 7.19–12.39) to 14.09 (9.96–25.52), Wilcoxon signed-rank test, *p* = 0.0156 in stages III and IV; 6.11 (2.29–6.46) to 7.03 (3.42–10.27), Wilcoxon signed-rank test, *p* = 0.1875 in stages I and II].

## Discussion

In this study, we found that surgical resection of the HNSCC tumors downregulated the plasma angiopoietin-1 level and upregulated the plasma angiopoietin-2 in conjunction with an increase in plasma NO level and reduced phosphorylation level of Tie2 in the erythrocyte membrane. Importantly, the angiogenic and NO signaling was significant only in stage III and IV HNSCC. The dynamic change of angiopoietin-Tie2 and NO signaling in the erythrocyte membrane in response to surgical trauma suggests that erythrocytes have an important role in modulating surgery-induced angiogenesis, which may provide an important implication for cancer surveillance and control.

Previous studies showed that surgical injury increases the plasma levels of angiopoietin-2 and VEGF in the lung, breast, and colorectal cancer [11, 12]. Our results further showed the plasma angiopoietin-2 was also upregulated by surgery in HNSCC. Angiopoietin-1 and angiopoietin-2 are two important cytokines and function with the vascular Tie2 receptor in regulating the complex process of angiogenesis [25, 26]. Accumulating evidences have demonstrated that like angiopoietin-1, angiopoietin-2 can induce the phosphorylation of Tie2 receptor and promote chemotaxis, tube formation, and sprouting of endothelial cells [4, 27]. The expression of angiopoietin-2 is upregulated at sites of tumor angiogenesis in multiple types of cancer, and overexpression of angiopoietin-2 promoted angiogenesis and tumor growth in experimental models [8, 28, 29]. Importantly, overexpression of angiopoietin 2 accelerated the carcinogenesis of oral squamous cell carcinoma through promoting epithelial-mesenchymal transition-induced angiogenesis [8]. Angiopoietin-2 has been proposed as a potential target for antiangiogenic drug development [30]. In our study, the increased angiopoietin 2 in plasma after surgery suggests that surgical injury may trigger the release of proangiogenic factors and facilitate the proliferation and metastasis of residual tumor cells in HNSCC [1, 11]. These results provided important evidence for identifying patients with a high risk of cancer relapse and establishing individualized anti-cancer therapy after surgical resection. However, current practice about surgical resection of primary tumors in HNSCC should not be changed until more evidence is gained to clarify the clinical impact of this angiogenic response on cancer prognosis.

The discrepancies of angiogenic expression in tumor cells between angiopoietin-1 and angiopoietin-2 have been reported in multiple types of cancer, including oral cancer [9], gastric cancer [31], and colon cancer [32]. The study has shown that compared with the healthy oral mucosa, angiopoietin-2 is overexpressed and angiopoietin-1 is downregulated in tumor samples of oral squamous cell carcinoma [9]. We proposed that the distinct response to surgery between angiopoietin-1 and angiopoietin-2 in our study may be explained by the mechanism that surgical manipulation of tumors induces the opposite regulation of angiopoietin-1 and angiopoietin-2 in the setting of surgical trauma.

Our results showed patients with HNSCC have a higher plasma level of NO before surgery compared to non-cancer controls. Besides, the raised plasma NO level in cancer patients was further enhanced after surgical resection of tumors. The study has showed that NO production induced by ethanol and tobacco may initiate an inflammatory response, dysregulate antioxidant protection system, and contribute to tumor growth in head and neck cancer [33, 34]. Investigators have showed surgical trauma stimulates the release of angiopoietin-2 and VEGF in plasma and promotes the process of angiogenesis in the lung, breast, and colorectal cancer [11, 12]. Our results further suggested a novel mechanism of surgery-induced angiogenesis via NO signaling pathway. NO has been shown to regulate the process of angiogenesis and promote tumor progression through the mechanism of vessel dilatation by eNOS, release of VEGF, activation of cyclooxygenase-2 stimulating the production of proangiogenic factors, and increased production of prostaglandin E2 inducing tumor vasculature hyperpermeability [10]. NO may also have clinical significance as a biomarker of inflammation and risk stratification of malignant transformation in patients with oral pre-cancer [10]. Researchers have proposed NO as a novel potential therapeutic target in resistant cancer by sensitizing cancer cells to chemotherapy and immunotherapy [35]. However, further studies are needed to evaluate the therapeutic applications of NO in cancer.

In this study, we found the baseline phosphorylation level of Tie2 in the erythrocyte membrane was significantly higher in cancer patients compared with non-cancer controls and was correspondingly reduced after surgery in connection with the decreased angiopoietin-1 level and increased angiopoietin-2 level in plasma. Tie2 is a receptor tyrosine kinase expressed principally on vascular endothelium and interacts with its ligand angiopoietin-1 and angiopoietin-2 in regulating vessel branching and maintaining endothelial homeostasis [36]. Experimental study recently demonstrated that an in vitro treatment with angiopoietin-1 for Tie2-overexpressed oral squamous cell carcinoma cells enhances the cell-cell and cell-extracellular matrix adhesive activities of cancer cells [37]. This finding indicates that angiopoietin-1 directly upregulates the functional activity of Tie2. In our study, the angiopoietin-1 plasma level was decreased after surgical resection, which may subsequently suppress the tyrosine phosphorylation level of Tie2 in the erythrocyte membrane. Our prior study has showed that osmopressor response activates the Tie2/Akt/eNOS signaling pathway in erythrocytes and stimulates the secretion of angiopoietin-1 in plasma [14]. The present study further demonstrated surgical injury activates the angiopoietin-Tie2 and NO signaling axis in the erythrocyte membrane, indicating that peripheral erythrocytes may serve as a potential diagnostic and therapeutic target for cancer patients.

There are limitations to our study. First, our findings should be interpreted with caution due to the small patient sample. Second, we did not measure the long-term risk of cancer recurrence and mortality after surgical resection and therefore could not evaluate the relationship between our biological findings and clinical outcomes. Third, more studies are needed to clarify how Tie2/Akt/eNOS signaling pathway interacts between vascular beds and erythrocytes in surgical injury. Fourth, we did not measure the long-term change of angiogenic response (e.g., 30 days after surgery), which is important for postoperative adjuvant therapy [28]. Finally, we did not measure the postoperative changes of angiogenic factors in non-cancer controls; therefore, whether our results are applicable to surgical patients without cancer is unclear.

In conclusion, the dynamic change of angiopoietin-Tie2 and NO signaling in the erythrocyte membrane in response to surgical resection of HNSCC suggests that erythrocytes have a significant role in modulating surgery-induced angiogenesis, which may provide a novel marker for cancer surveillance and control. We need more studies to evaluate the relationship between changes in angiopoietin-Tie2 and NO expression on cancer outcomes after tumor resection and to explore potential clinical applications of angiopoietin-Tie2 and NO signaling in cancer prevention and treatment.

## Data Availability

The datasets used and/or analysed during the current study are available from the corresponding author on reasonable request.

## Abbreviations

AJCC: American Joint Committee on Cancer
EDTA: Ethylenediaminetetraacetic acid
eNOS: Endothelial NO synthase
HNSCC: Head and neck squamous cell carcinoma
IQR: Interquartile range
NO: Nitric oxide
PMSF: Phenylmethylsulfonyl fluoride
SCC: Squamous cell carcinoma
SD: Standard deviation
TNM: Tumor node metastasis
VEGF: Vascular endothelial growth factor
